# First person – Ling-shiang Chuang

**DOI:** 10.1242/dmm.041624

**Published:** 2019-08-13

## Abstract

First Person is a series of interviews with the first authors of a selection of papers published in Disease Models & Mechanisms (DMM), helping early-career researchers promote themselves alongside their papers. Ling-shiang (Felix) Chuang is first author on ‘Zebrafish modeling of intestinal injury, bacterial exposures and medications defines epithelial *in vivo* responses relevant to human inflammatory bowel disease’, published in DMM. Ling-shiang is an instructor in the lab of Judy Cho at Icahn School of Medicine at Mount Sinai, New York, USA, investigating how to establish human genetic-driven personalized drug treatments for inflammatory bowel disease (IBD) by using zebrafish as a screening model.


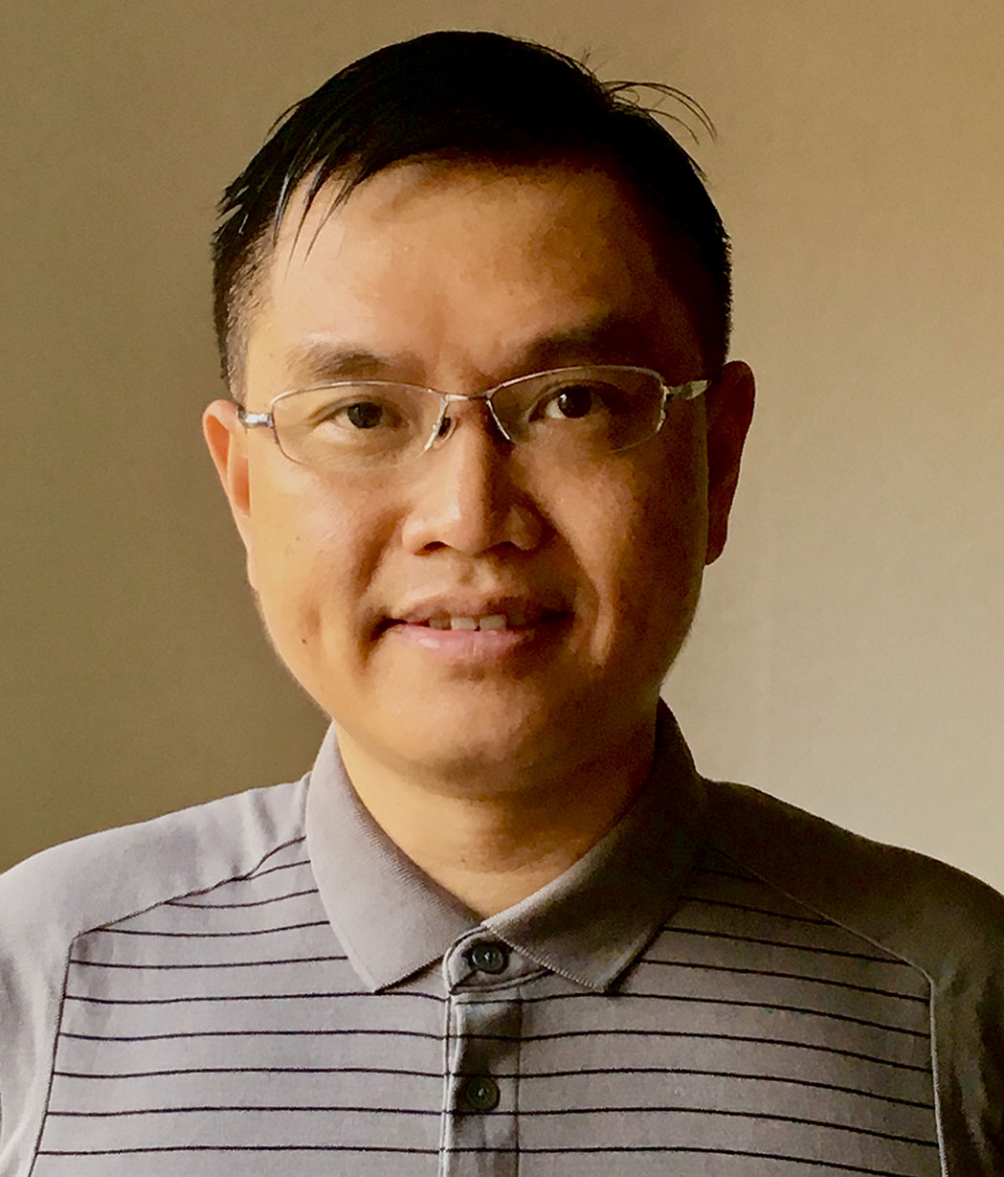


**Ling-shiang (Felix) Chuang**

**How would you explain the main findings of your paper to non-scientific family and friends?**

IBD is composed of two subtypes, Crohn's disease and ulcerative colitis, which are estimated to affect more than 3-million adults in the United States. IBD is a complex disease, defined by interactions of human genetics, host epithelial and immune response, the microbiome and environmental factors. In recent years, progress has been made in associating IBD phenotypes to genetics and genomic information of patients. Mutations in genes for bacterial sensing, bacterial cleaning and immune cell activation increase the risk of IBD. The common IBD therapeutic agents are still costly, and result in moderate to low responses. We realized that novel methods are required and turned to developing an IBD animal model to better understand the effectiveness of IBD therapeutic agents in patients with different genetics, host response and microbiome. In this paper, we built these zebrafish intestinal injury models to mimic and measure the effects of acute inflammation and chronicity, bacterial exposure and drug response. In the era of precision medicine, our models scale more rapidly for a sophisticated understanding of time course factors, disparate cellular effects and mechanisms of action for established and new drugs.

“These intestinal injury models will pave the way for human genetic-driven personalized medicine approaches for IBD patients.”

**What are the potential implications of these results for your field of research?**

Our zebrafish intestinal injury models provide a unique opportunity to advance the field's understanding of the interactions between genetics, host response, the microbiome and therapeutic agents. These interactions can be further dissected by time course factors, disparate cellular effects and mechanisms of action of therapeutic agents. These intestinal injury models will pave the way for human genetic-driven personalized medicine approaches for IBD patients.

**What are the main advantages and drawbacks of the model system you have used as it relates to the disease you are investigating?**

The large sample sizes feasible with zebrafish-based studies allow us to conduct careful, time-course analyses relative to injury. The superior speed, sample size, visualization (e.g. microbes, autophagy) and the key role of the epithelial barrier makes high-throughput studies in zebrafish an important component of the IBD therapeutic development pipeline. The only drawback is that all the discoveries in the zebrafish model still require validation in human cells or mice.

“The therapeutic agents of human IBD are effective in our zebrafish model.”

**What has surprised you the most while conducting your research?**

The therapeutic agents of human IBD are effective in our zebrafish model. These reagents can either work by reducing the severity of the injury, or promote mucosal healing. These data give us hope that the mechanisms of action of the new therapeutic agents observed with screening in our models can be applied back directly to human IBD patients in the future.

**Figure:**
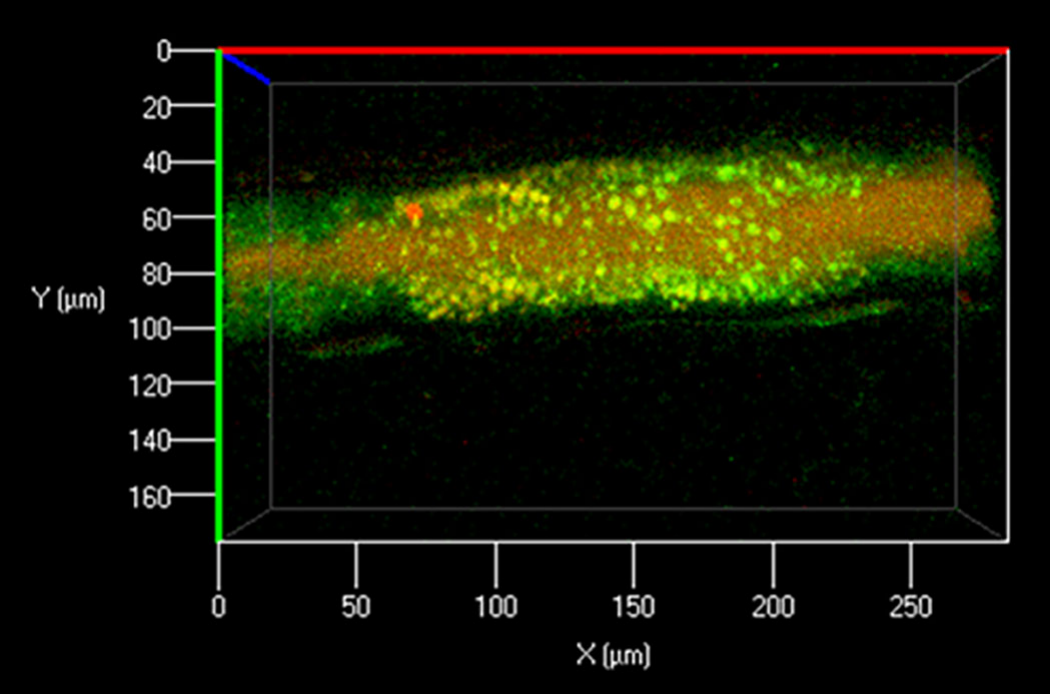
**3D reconstruction of zebrafish posterior mid-intestine with endocytosed *E. coli* proteins and autophagy.** Endocytosed *E. coli* proteins were labeled with pHrodo (red). Autophagosomes were labeled with Cyto-ID (green).

**Describe what you think is the most significant challenge impacting your research at this time and how will this be addressed over the next 10 years?**

Genetically speaking, complex diseases like IBD are the result of polygenicity. The polygenic risk scores have been used in estimating the disease risk for individuals based on their risk variants profile. However, the concept of combining polygenic risk is a big challenge for any mechanistic, or functional, validation studies using animal disease models, at this time. Hopefully, within a decade, we can make some breakthroughs on high-throughput gene/genome editing, and combine this with large-scale profiling of single-cell transcriptome and proteome to build disease animal models to functionally address the polygenicity of human diseases.

**What changes do you think could improve the professional lives of early-career scientists?**

We need to encourage, support and provide funding for early-career scientists to develop new models, methods and technologies. Also, rather than mostly promoting and focusing on well-established principal investigators, with more complete stories, early-career scientists need help with exposure in traditional networking and social media. I am especially thankful to the Disease Models & Mechanisms journal for giving me this opportunity to promote my novel work.

**What's next for you?**

I am currently looking for a tenure track faculty position to apply and expand my knowledge of IBD, zebrafish modeling and human mucosal immunology.
